# Using Smart Speaker Technology for Health and Well-being in an Older Adult Population: Pre-Post Feasibility Study

**DOI:** 10.2196/33498

**Published:** 2022-05-09

**Authors:** Rachel McCloud, Carly Perez, Mesfin Awoke Bekalu, K Viswanath

**Affiliations:** 1 Dana-Farber Cancer Institute Boston, MA United States; 2 Harvard T H Chan School of Public Health Harvard University Boston, MA United States

**Keywords:** technology, older adults, communication inequalities, digital health, elderly population, smart technology, smart speaker, well-being, health technology, mobile phone

## Abstract

**Background:**

Although smart speaker technology is poised to help improve the health and well-being of older adults by offering services such as music, medication reminders, and connection to others, more research is needed to determine how older adults from lower socioeconomic position (SEP) accept and use this technology.

**Objective:**

This study aimed to investigate the feasibility of using smart speakers to improve the health and well-being of low-SEP older adults.

**Methods:**

A total of 39 adults aged between 65 and 85 years who lived in a subsidized housing community were recruited to participate in a 3-month study. The participants had a smart speaker at their home and were given a brief orientation on its use. Over the course of the study, participants were given weekly check-in calls to help assist with any problems and newsletters with tips on how to use the speaker. Participants received a pretest and posttest to gauge comfort with technology, well-being, and perceptions and use of the speaker. The study staff also maintained detailed process notes of interactions with the participants over the course of the study, including a log of all issues reported.

**Results:**

At the end of the study period, 38% (15/39) of the participants indicated using the speaker daily, and 38% (15/39) of the participants reported using it several times per week. In addition, 72% (28/39) of the participants indicated that they wanted to continue using the speaker after the end of the study. Most participants (24/39, 62%) indicated that the speaker was useful, and approximately half of the participants felt that the speaker gave them another voice to talk to (19/39, 49%) and connected them with the outside world (18/39, 46%). Although common uses were using the speaker for weather, music, and news, fewer participants reported using it for health-related questions. Despite the initial challenges participants experienced with framing questions to the speaker, additional explanations by the study staff addressed these issues in the early weeks of the study.

**Conclusions:**

The results of this study indicate that there is promise for smart speaker technology for low-SEP older adults, particularly to connect them to music, news, and reminders. Future studies will need to provide more upfront training on query formation as well as develop and promote more specific options for older adults, particularly in the area of health and well-being.

## Introduction

### Background

As the global population ages, new solutions that address multiple dimensions of health and well-being are needed to ensure healthy aging. In addition to promoting physical health, a range of factors may contribute to emotional and social well-being, which are key pillars for healthy aging and the ability of older adults to lead rich, independent lives [1]. Those from lower socioeconomic position (SEP) often face heightened challenges grappling with the environmental demands of maintaining autonomy because of poor health and mobility and are often at greater risk for isolation and lower psychological well-being than their higher SEP counterparts [2]. However, the ways in which protective factors may be leveraged to contribute to healthy aging are often underemphasized.

Factors that may help low-SEP older adults compensate for limitations or lack of resources or may help them leverage their positive capabilities and interests may be vital to promoting and maintaining well-being [2]. Information and communication technologies are low-cost, innovative resources that allow older adults to facilitate and maintain a connection with the outside world and improve psychological, social, and physical well-being [3].

Previous research has indicated that there is an association between technology use and well-being among older adults, with internet use reducing loneliness, predicting better mental health, increasing life satisfaction, and improving communication [4,5].

Technology offers the opportunity to increase connections with friends and family and connect to the necessary resources and knowledge (such as providing links to news sources or services) to remain engaged with the society at large without in-person interactions with others [3]. In addition to the connection to others, the ability of technology to link older adults to interests such as music can also foster greater emotional well-being. In light of the COVID-19 pandemic, the potential of technology to help alleviate loneliness has become even more salient [6].

However, several challenges have historically impacted the ability of older adults to fully engage with technology [7]. For example, vision issues may make it difficult for older adults to see screens, and dexterity challenges may impact the ability to use a keyboard or mouse. Even during the COVID-19 pandemic, when in-person activities were limited, older adults were less likely to say that the internet was an essential part of life compared with younger adults [8]. Literacy issues may inhibit the ability to read what is presented on the screen. These issues may be further exacerbated in low-SEP older adults, particularly those who are unable to afford adequate care to address vision or dexterity issues [7]. Further challenges for low-SEP individuals include decreased access to internet services, cutting them off from a crucial channel of health information and well-being resources [9]. This may help fuel communication inequalities or the differences in access to, understanding of, and acting upon health information [10], which may impact which older adults are able to benefit from technology. Such differences could create disparities, as some groups are more readily able to engage with technology to access health information and resources compared with others. Voice interface technology, such as the Amazon Echo or Google Home, shows great promise in reducing social isolation and assisting in healthy aging. The voice technology uses a zero-user interface design in which users engage with a smart speaker through voice commands. This technology works without screens and keyboards to create a system that is more accessible to older adults, as it removes barriers related to vision, dexterity, or literacy [11] and may thus reduce barriers to technology use that disproportionately impact low-SEP older adults. These interfaces also allow for the use of natural communication (speaking) instead of navigating and scrolling through webpages or learning how to operate new technology.

A key component of this interface is the feature of voice-activated personal assistants, which use artificial intelligence to create tailored, personal interactions through adaptive learning systems that may converse directly with the user in a back-and-forth discussion [11]. In addition to providing a simple way to engage with the speaker, it is proposed that the voice-activated personal assistants feature may also provide companionship and entertainment to older adults, providing the ability to alleviate loneliness and increase psychological well-being [11]. Reminders offered through the system may also help improve health behaviors such as medication adherence [12]. Voice interfaces provide a rich set of services through their *skills*, which are voice-based apps (similar to voice-based versions of apps that appear on smartphones). Commonly used skills include the ability to play music, find recipes, check the news, and ask health-related questions.

Although there is promise in enhancing well-being through these devices, research involving smart speakers and older adults is still in its early stages. Often, research involving smart speakers is conducted using one-time quantitative or qualitative surveys that gauge potential interest or initial reactions. A feasibility study conducted in a California retirement community deployed smart speaker devices in residents’ homes, conducting focus groups and training workshops [13]. Feedback from participants indicated high satisfaction with technology, with 75% of participants reporting daily use. Participants felt that the speaker helped them stay connected with the community, family, and the digital world [13,14].

However, it is vital to replicate and expand these studies with more groups of older adults to truly gauge their acceptance and relevance for this age group. Although the features of voice technology are poised to remove barriers to traditional technology use, more research is needed on the exact features of the technology that are perceived as most beneficial for older adults and the areas in which they require additional learning to use the speaker successfully. Furthermore, research should focus on low-SEP individuals, particularly given the previous findings of challenges with other forms of technology, to ensure that using these smart speakers does not further propagate communication inequalities. Measuring the potential barriers and facilitators of speaker use can determine whether these speakers will resonate with older adults in ways that can foster health and well-being.

### Objectives

This study aimed to investigate the feasibility of using voice interface technology in group of low-SEP older adults living in a subsidized residence for older adults. Our specific aims included (1) documenting the frequency of use of technology and the ways in which the technology was used; (2) assessing the acceptability and usability of the technology, as measured through process data and survey assessment; and (3) measuring the interest in and the use of the speaker for health information seeking.

## Methods

### Ethics Approval

Older adults were recruited from a residence facility for older adults subsidized by the United States Department of Housing and Urban Development (HUD) in South Georgia to participate in a 3-month feasibility study. This study was reviewed and approved by the Harvard T.H. Chan Institutional Review Board (19-0304).

### Recruitment

**Figure 1:**
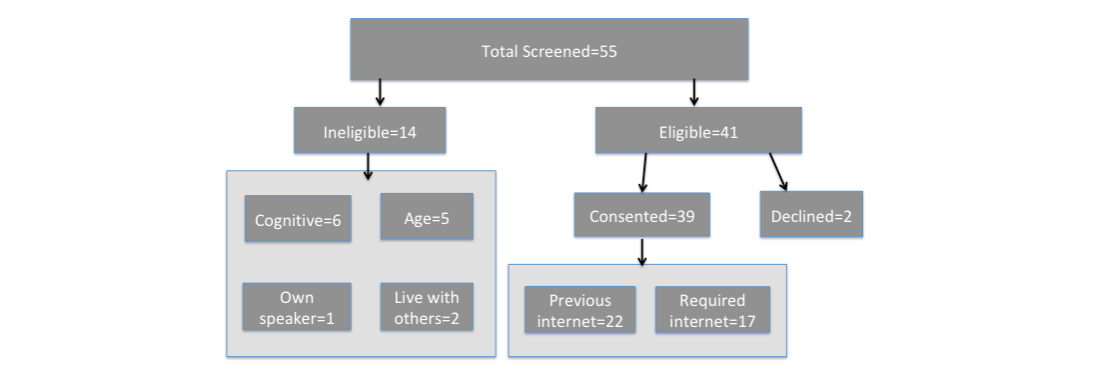
Participant flowchart.

The eligibility criteria for the study included being in the age range of 62-85 years, living alone, and not having a smart speaker currently at their residence. The upper bound of 85 years was determined because of the increased risk of cognitive concerns such as memory loss or dementia past this age. The site principal investigator made an initial presentation to a group of 52 residents. In the presentation, she presented the features of the smart speaker and the details of the study through a Microsoft PowerPoint presentation. At the end of the presentation, interested residents were invited to fill out a screening form for consideration for joining the project. The names of those who appeared eligible were discussed with the center staff to determine if they were cognitively able to participate. There were 24 individuals who filled out the screening form after the presentation; however, only 15 individuals were eligible. There were 11 individuals who filled out the screening form but were ineligible: 3 individuals were excluded because of cognitive concerns, 5 individuals were excluded because of age >85 years, 2 individuals were excluded because of living with others, and 1 individual was excluded because of already owning a smart speaker (Figure 1).

Although many participants expressed hesitance to adopting a new technology, there were several factors that helped to overcome this hesitance. The first was the partnership with and advocacy of the management staff at the residence in helping to recruit and reassure residents. Throughout the study, they served as champions for the project and helped to encourage residents to participate. They also kept a speaker unit in their office to learn about the speaker and to demonstrate its use to the residents. Second, as the first participants were connected to their speakers, other residents became interested after seeing the participants demonstrate the speaker’s capabilities. Once the first residents provided positive reports on their speaker use, more residents agreed to participate. Therefore, we created a rolling enrollment process that spanned 2.5 weeks to recruit and enroll the remaining participants, reaching a final number of 39 participants (Figure 1). During this time, 3 additional residents were deemed ineligible because of cognitive concerns, and 2 eligible participants declined to participate.

Screened, eligible participants were then walked through the consent process by the study team. Before signing the consent form, each participant reviewed the terms of use of the smart speaker with the site principal investigator and then discussed a set of comprehension questions to ensure that participants understood the basics of the smart speaker (such as data tracking and privacy) before signing. After signing the terms of use of the speaker, the site principal investigator reviewed all study details with the participant and then had them sign the study consent form.

Before installing the smart speaker, the research team verified that each resident had internet access in their apartment. Overall, 56% (22/39) of the participants had internet access in their apartment before the study. Those without internet connection were provided connection through either a 3-month account setup and paid for by the study staff or assistance to obtain a low-cost internet connection offered for HUD housing–based residents in their own name. Most participants (14/17, 82%) elected to set up their own internet accounts.

Once internet connectivity was established or verified, the study staff visited the participant’s residence to install the smart speaker in the home. Each participant’s speaker was connected using their unique study account ID and study-generated email address. After the speaker was connected and activated, the staff provided the participant with a brief orientation to the speaker, including how to ask questions to the unit, adjust the volume, play music, and set reminders and alarms. In addition to the brief introduction, each participant received an introductory sheet on the smart speaker that provided instructions on how to make simple queries or give commands.

The participants were then involved in the study for a 3-month period. During the study, participants received weekly check-ins with study staff in the form of phone calls and help desk hours. All issues were documented by the study staff and addressed as needed. The site principal investigator held 3 workshops throughout the study period in which participants’ questions were answered and additional tips were shared on how to use the speaker. Periodic newsletters introduced other ways to use the device, providing wording to request that the speaker perform actions such as reading Bible verses, tracking calories, and playing games. Emphasis was placed on activities that increased health (connecting to services such as WebMD or calorie trackers) or mental well-being (learning to have conversations with the speaker and connecting to podcasts or games).

At the end of the study, if the participant desired to continue with the speaker, the study staff assisted them in connecting the speaker to their personal accounts. If the participant did not want to continue using the speaker, the study staff assisted them with deregistering the speaker and canceling internet accounts, if needed.

### Data Collection

Once participants consented to participate in the study, they received a paper-based pretest survey that asked them about their mental well-being, loneliness, social well-being, demographics, prior technology use, comfort with technology, and social networks.

During the 3-month study period, participants’ speaker use data were recorded through their study account; each query made to the speaker was documented in the speaker’s records. As a user operates the smart speaker, each interaction with the speaker is recorded in the user’s personal account. One key component of the study was the use of back-end data to record the interactions with the device. To do so, the study staff created study-related email accounts and study-related accounts with the speaker’s parent company. These accounts were created so as not to use participants’ real names in any recording; the study accounts only contained links to the participant’s ID number. Process data were also tracked through a spreadsheet in which the study staff recorded each weekly check-in call with the participants, including any specific comments that were made.

At the end of each participant’s 3-month study period, the participant was given a paper-based posttest that asked for repeated measures on health and well-being as well as asked questions on usability and perceptions of the speaker.

### Measures

#### Pre- and Posttest Measures

Responses from the paper-based pre- and posttest survey, along with the process data gathered throughout the survey, were included in this study.

#### Sociodemographics

The participants were asked about their age, sex, income, and highest level of education. Food security was measured by providing the statement, “in the past 12 months, the food we bought ran out and we did not have money to get more,” with response options being never, sometimes, or often true. Income security was measured by asking participants to select a phrase that described their household income, with options being living comfortably, getting by, finding it difficult, or finding it very difficult on present income.

#### Current Use of Technology

We asked participants if they had ever used the internet, how often they used it, and if they had internet access at their residence. Participants were asked to select the devices they currently owned and used, with response options including smartphone, tablet, computer, and smart television. Participants were also asked to indicate their level of comfort with technology, with response options including not comfortable at all, somewhat comfortable, comfortable, and very comfortable using technology.

#### Perceived Usability of the Speaker

In the posttest, participants were first asked how often they used the speaker during the 3-month study period. They were then asked to rate whether the speaker was easy to use, if it seemed easier to use over time, and if they understood how to ask the speaker a question, with response options ranging from totally disagree to totally agree. Participants then rated how often the speaker correctly understood their questions, with response options ranging from all the time to never.

#### Reactions to the Speaker

On the basis of the feedback gathered throughout the study, we created an index of reactions to the speaker content, including how connected the speaker made them feel to the world, if it helped them keep track of appointments, if the speaker helped them keep track of the day or time and appointments, and if it made them feel less lonely.

### Process Data Notes

A spreadsheet was created that tracked each interaction with the participants, including phone calls and in-person interactions. For each interaction, the study staff listed the topic of the call, including any issues or positive statements about the speaker. Resolutions for these issues were also noted.

### Analysis

Responses to the pretest and posttest were analyzed. Frequencies and percentages were gathered from all pretest and posttest variables. Process notes were analyzed for counts of certain types of issues or interests expressed by the participants.

## Results

### Overview

The final sample comprised 39 participants (Table 1). Most (36/39, 92%) of the participants were female and White (38/39, 97%). The demographics of the study participants closely matched the demographics of the community. All the participants had an income of ≤US $35,000, with the majority having an income of <US $15,000 per year. Approximately half (20/39, 53%) of the participants felt that they were getting by on their current income, although 34% (13/39) of the participants indicated that they sometimes or often experienced food insecurity.

Before the study, 56% (22/39) of the participants were using the internet (on a computer or smartphone) at least once per day, and 33% (13/39) of the participants rarely or never used the internet, with those who had in-unit internet access significantly more likely to use the internet frequently (Table 2; χ^2^=11.6; *P*=.02). The most frequently owned device was a smartphone (21/39, 54%).

**Table 1:** Participant demographic characteristics (n=39).

Demographic		Values
**Sex, n (%)**		
	Female	36 (92)
	Male	3 (7)
Age (years), mean (SD; range)		72.62 (5; 64-82)
**Race, n (%)**		
	White	38 (97)
	Black	1 (2)
**Income (US $), n (%)**		
	0-9999	11 (29)
	10,000-14,999	15 (40)
	15,000-19,999	8 (21)
	20,000-34,999	3 (8)
**Education, n (%)**		
	<8 years	1 (2)
	8-11 years	5 (12)
	12 years or completed high school	5 (12)
	Post high school training other than college (vocational or technical)	9 (23)
	Some college	10 (25)
	College graduate	5 (12)
	Postgraduate	2 (5)
**Income security, n (%)**		
	Living comfortably on present income	6 (15)
	Getting by on present income	21 (53)
	Finding it difﬁcult on present income	10 (25)
	Finding it very difﬁcult on present income	2 (5)
**Food ran out and did not have money to buy more, n (%)**		
	Never true	26 (66)
	Sometimes true	12 (30)
	Often true	1 (2)

**Table 2:** Technology use before the study (n=39).

			Values, n (%)
**Frequency of internet use**			
	Never	9 (23)	
	Rarely	4 (10)	
	Once or twice a month	0 (0)	
	Once a week	0 (0)	
	A few times per week	2 (5)	
	Once a day	3 (7)	
	Many times per day	19 (48)	
Had internet access at their residence before the study			22 (56)
**Devices owned**			
	Smartphone	21 (55)	
	Tablet	17 (44)	
	Computer	11 (28)	
	Smart television	6 (15)	
	None of the above devices owned	8 (21)	
**Comfort with using the internet**			
	Not comfortable at all	5 (12)	
	Somewhat comfortable	13 (33)	
	Comfortable	10 (25)	
	Very comfortable	11 (28)	

### End of Study Device Uptake

By the end of the study period, 38% (15/39) of the participants said that they had used their speaker once per day, and 38% (15/39) of the participants had used it several times per week. There were no significant differences between having internet access before the study and use or usability ratings of the smart speaker.

After the study, 72% (28/39) of the participants kept the speaker, with 53% (9/17) of the participants who did not have prior access keeping the speaker and retaining their internet access to continue use. Of the 11 participants who did not keep their speaker (Textbox 1), 3 had to do so because of their inability to maintain their internet connection. One cited increased concerns about privacy. Another stated that the speaker had given her the confidence to get the internet and try new things but that she felt that she got more value from a newly purchased tablet.

**Reasons given for terminating speaker.**

**Reason given by participants**

1. Relied on a study-based internet connection and were not able to maintain internet connection. (3 participants)
2. Had increased privacy concerns and did not want to use the speaker (1 participant)
3. Felt that other newly purchased devices (tablet) provided more value (1 participant)
4. Had trouble hearing the speaker and lost interest (1 participant)
5. Had difficulty with the internet provider and lost interest (1 participant)
6. Did not have any other devices; did not want to maintain the internet connection (1 participant)
7. Liked the device but did not want to continue using it (1 participant)
8. Did not use the device often and did not want to maintain the internet connection (1 participant)
9. No reason given (1 participant)

### Self-reported Uses of the Speaker

Common uses of the speaker that were quantified in the posttest are reported in Table 3, with all participants (39/39, 100%) citing that they used the speaker for the weather and 89% (34/39) of the participants saying that they used the speaker to listen to music. On reviewing the process data, we found that participants cited additional uses in their conversations with study staff, including using the speaker to read Bible verses, relying on the unit for reminders (such as for medication or physician appointments), meditation, and asking about the date and time. Another finding noted in the process data notes was that participants enjoyed saying *good morning* and *good night* to the unit.

**Table 3:** Percentage of participants using the speaker for various activities (n=38).

Activity	Values, n (%)
Weather	38 (100)
Music	34 (89)
Health	21 (55)
News	17 (44)
Conversation	17 (44)

### Reactions to the Speaker

More than half of the participants agreed or strongly agreed that the speaker was useful (24/39, 63%), helped them obtain the required information (24/39, 62%), and helped them keep track of the day and time (24/39, 62%; Table 4). Approximately half of the participants said that the speaker provided another voice to talk to (19/39, 49%) and felt that the speaker made them feel more connected to the world (18/39, 46%). Although almost half of the participants (17/39, 44%) felt that the speaker reconnected them to interests such as music or history, fewer participants (9/39, 23%) reported that the speaker helped them with their health.

**Table 4:** Reactions to the speaker (n=39).

Speaker reaction	Strongly disagree or disagree, n (%)	Neutral, n (%)	Agree or strongly agree, n (%)
Having a speaker made me feel more connected with the world.	4 (10)	16 (41)	18 (46)
Having a speaker makes me feel more confident about using technology.	5 (12)	15 (38)	19 (48)
Having a speaker gave me an opportunity to strengthen my relationships at the Towers.	8 (20)	22 (56)	8 (20)
Having a speaker made me feel closer to my family (children, grandchildren).	10 (25)	19 (48)	7 (17)
The speaker made me feel like I had another voice to talk to.	8 (20)	10 (25)	19 (48)
The speaker helped me keep track of what day or time it was.	4 (10)	10 (25)	24 (61)
The speaker made it easier to get the information I need.	2 (5)	11 (28)	24 (61)
The speaker helped me keep track of my commitments and appointments.	2 (5)	20 (51)	15 (38)
The speaker made me feel less lonely.	8 (20)	19 (48)	9 (23)
The speaker provided me with a sense of comfort.	9 (23)	19 (48)	9 (23)
By the end of the study period, the speaker interactions were important to me.	5 (12)	16 (41)	18 (6)
The speaker reconnected me to my interests (such as music, art, or history).	6 (15)	15 (38)	17 (43)
The speaker helped me with my health or nutrition.	11 (28)	17 (43)	9 (23)
I felt like there was something lacking in my interactions with the speaker.	13 (32)	18 (48)	7 (19)
The speaker met my expectations.	5 (13)	15 (40)	17 (45)
The speaker was useful to me.	2 (5)	12 (31)	24 (63)

### Process Data Findings and Responses

During the early weeks of the study, 31% (12/39) of the participants commented in their check-in calls about the difficulties with asking questions to the unit. Owing to frequent comments about this issue, a workshop was held to provide assistance. Within this workshop, it was revealed that there were the following concerns: (1) participants felt that the device was giving inconsistent answers to the same question, (2) participants felt that the device was not acknowledging them, and (3) participants were often asking questions that were too long or complex (such as multipart questions). Many participants were asking multipart questions that the speaker could not process, were pausing too long between the wake word and their request, or were spending time trying to frame their request in a polite manner (during which time the speaker determined that the request was not relevant). Within this trial session and workshop, it was also determined that there were some options that required skills (particular apps that are saved within the speaker preferences only once the user activates them) to provide reliable answers to particular questions. For example, if the participant wanted the speaker to read Bible verses, a particular skill could be enabled that would provide the desired results of requesting a particular verse. However, without requesting to open this skill, the speaker may pull from various other sources (or interpret their request as asking the speaker to recite the whole Bible from the beginning), thus the answer discrepancy. Owing to these issues, the research team offered suggestions and created a newsletter that addressed common ways of wording (such as focusing on brief, direct commands) and simple directions to enable commonly desired skills.

Another issue discovered through process notes was that 5 participants had volume issues. Volume issues were often exacerbated by loud air conditioner units that were in the same room as the speaker, as most participants preferred that the speaker be placed in their living room. Each participant who noted a volume issue was provided with an additional Bluetooth speaker that could increase volume.

Within the process notes, several direct quotations noted also speak of the positive reaction to the speaker (Textbox 2).

**Reactions to the speaker.**

**Quotes from participants about their speaker interactions**

- “it is nice to have someone to talk to.”
- “it’s like [the speaker] is a little friend.”
- “I enjoy the company.”
- “The speaker has ‘become part of the family’.”
- “at least I have somebody to talk to.”

## Discussion

### Principal Findings

This feasibility study indicates promise for the use of smart speaker technology in a group of low-SEP older adults. By providing the speaker and related support to older adults, the research team was able to observe the barriers and facilitators of initial speaker use in this population. Overall, the speaker was well received by most of the sample, with 38% (15/39) reporting daily speaker use and 38% (15/39) reporting using it several times per week. Participants perceived the speaker as useful and assisting them in finding the information they needed. Although there were initial barriers to uptake in using the speaker, frequent monitoring and check-ins with study staff alerted the study staff to these issues; once addressed, the perceived ease of use of the speakers appeared to increase.

Notably, most of the participants (28/39, 72%) opted to continue using the speaker after the study period ended. This illustrates the journey of many participants from being technology hesitant to routinizing technology use in their day-to-day lives. Although there was a learning curve for understanding how to frame questions to the speaker, it was embraced by many respondents. At the end of the study, 53% (9/17) of the participants who did not have internet access before the study elected to maintain internet access and continue using the speaker. This echoes the patterns observed by the Pew Research Center that once older adults are on the web, the technology becomes a daily fixture; among internet users aged ≥65 years, 75% use the internet on a typical day at least daily, with 51% saying they go on the web several times per day and 8% say they use the internet almost constantly [15]. This percentage has increased among smartphone users, with 76% of those owning a smartphone using the internet several times per day or more.

Participants found many sources of value of the speaker, including connecting to the world, accessing information, and using the device for reminders. Process data notes taken during check-ins reflect the interest in using the speaker for reminders of doctors’ appointments and social engagements, particularly to remember events happening within the residence. Participants indicated the value of the speaker to help them keep track of the day and time, a feature that was deemed useful for keeping track not only of appointments but also to anchor them to the present. The frequency and enthusiasm with which participants used the speaker for this purpose and for connecting to music and other interests suggests the potential for this technology to be an asset to this population, particularly in overcoming some of the additional challenges to mobility, dexterity, and resource access by low-SEP older adults [2]. For example, the connection participants felt to music and other interests through the speaker was an integral part of facilitating interest and continued use and may also connect to emotional well-being. Future analyses will explore the back-end data of the device to determine in detail the frequency and variety of use of the speaker.

This study identifies some potential strengths and weaknesses of smart speakers in this population. Despite the initial hesitance with the speaker, most participants found value in the speaker, using it several times per week, with many even reaching daily use. Future analyses of our back-end data will further explore how this has been integrated into their lives.

This study also points to potential issues that should be addressed when designing a smart speaker study within this population. First, initial hesitance to technology use may be noted, particularly because of privacy concerns. Within this project, the buy-in and assistance of the staff at the residence were critical to project success, as they provided space for residents to convene for speaker-related activities and served as champions for our process. Second, this study points to the need for targeted training on using the device once it is installed, with emphasis placed on how to properly craft questions and how to activate certain skills of interest. Of note is the lower percentage of participants (9/39, 23%) who felt that the speaker helped them with their health or nutrition. Within the study, the newsletters that were provided to participants emphasized different health-related activities for participants to try, such as asking for calorie information in certain foods but process findings suggest that these queries did not always yield useful information. These frustrations with correctly framing questions have also been cited in a previous qualitative study, in which groups of older adults were invited to test out the speaker [16]. Studies that intend to use a smart speaker for health-related purposes should be prepared to provide direct training on how to ask questions about health-related topics or should train residents to use a selected health-related skill (such as a specific nutrition app). Further benefit may be gained by orienting the user to skills that are specifically created to address the needs of older users [16]. Furthermore, in this study, we noted that several participants had difficulty in hearing the speaker. We tested a smaller version of the speaker; the full-size unit with a larger speaker may be required as well as the option to receive a Bluetooth speaker that can reach a higher volume.

This study had some limitations. As we created study accounts, we were not able to sign participants up for some of the specific older adults–focused skills that required passwords and personal details; therefore, we were unable to study the use of these targeted apps. Our recruitment also occurred within one senior housing unit in which participants would often share tips and experiences with other participants in the study. It is unknown whether we would have had the same success if we had conducted this study with home-dwelling older adults.

Despite these challenges, smart speakers are still poised to alleviate some of the main barriers to technology use in this population once these factors are addressed. Many functions that were of value to the participants were integrated quickly into routines upon brief demonstration (playing music, setting reminders, and the asking for time) and showed immediate value at a low cost. Several participants reported gaining confidence in technology because of the ease of the speaker for basic functions, and they were eager to learn how to explore more uses. Speaker manufacturers may consider building in features that can aid in understanding additional speech patterns and have easier-to-navigate menus of information options built into basic features. Although some of these features may appear in certain skills geared toward older adults, these functions would assist a larger section of the population that has low literacy and low health literacy use the unit to find the required information. Future research may explore in greater detail the value of other aspects of the speaker, such as the aforementioned specific skills designed for older adults as well as other smart devices that can be linked to the speaker to assist further with daily life, such as smart switches and thermostats, that may assist low-SEP adults in living independently.

### Conclusions

Smart speakers show great promise for providing low-SEP older adults with an opportunity to increase their connection with music, news, and the weather as well as providing a way to anchor them to the date and time. As research using this speaker in this population progresses, special attention should be paid to the design of health-related skills and services to determine the best way to engage older adults with relevant, useful health content.

## Abbreviations

SEP: socioeconomic position
